# Green Perylene Bisimide Dyes: Synthesis, Photophysical and Electrochemical Properties

**DOI:** 10.3390/ma7085488

**Published:** 2014-07-25

**Authors:** Che-Wei Chang, Hsing-Yang Tsai, Kew-Yu Chen

**Affiliations:** Department of Chemical Engineering, Feng Chia University, Taichung 40724, Taiwan; E-Mails: m0111617@fcu.edu.tw (C.-W.C.); p0156676@fcu.edu.tw (H.-Y.T.)

**Keywords:** green dyes, 1-(*N*,*N*-dialkylamino)perylene bisimides, intramolecular charge transfer, solvatochromism, Lippert-Mataga equation, density functional theory calculations

## Abstract

Three asymmetric amino-substituted perylene bisimide dyes with different *n*-alkyl chain lengths (*n* = 6, 12, or 18), 1-(*N*,*N*-dialkylamino)perylene bisimides (**1a**–**1c**), were synthesized under mild condition in high yields and were characterized by ^1^H NMR, ^13^C NMR (nuclear magnetic resonance), HRMS (High Resolution Mass Spectrometer), UV-Vis and fluorescence spectra, as well as cyclic voltammetry (CV). These molecules show intense green color in both solution and solid state and are highly soluble in dichloromethane and even in nonpolar solvents, such as hexane. The shapes of the absorption spectra of **1a**–**1c** in solid state and in solution were found to be virtually the same, indicating that the long alkyl chains could efficiently prevent aggregation. They exhibit a unique charge transfer emission in the near-infrared region, of which the peak wavelengths show strong solvatochromism. The dipole moments of the compounds have been estimated using the Lippert-Mataga equation, and upon excitation, they show larger dipole moment changes than that of 1-aminoperylene bisimide (**2**). Furthermore, all of the compounds exhibit two quasi-reversible one-electron oxidations and two quasi-reversible one-electron reductions in dichloromethane at modest potentials. Complementary density functional theory (DFT) calculations performed on these dyes are reported in order to rationalize their molecular structures and electronic properties.

## 1. Introduction

Perylene bisimides (PBIs) and their related derivatives have continuously received significant attention due to their potential applications in molecular optoelectronic devices, such as organic field-effect transistors (OFETs) [1,2,3,4,5,6], photovoltaic cells [7,8,9,10,11,12,13,14,15,16], light-emitting diodes [17,18,19,20,21], light-harvesting arrays [22,23], photochromic materials [24,25], molecular wires [26,27] and LCD color filters [28,29]. In addition, PBIs have been used as building blocks to construct supramolecular or artificial photosynthetic systems [30,31,32,33]. These organic dyes are advantageous, due to their high molar absorptivities, reversible redox properties and optical stabilities, ease of synthetic modification and excellent thermal stability [34,35,36,37,38,39,40,41,42,43,44,45,46,47,48,49,50,51,52,53,54,55]. The electronic characteristics of PBIs can also be fine-tuned by the substitution of the conjugated aromatic core. Based on these principles, many perylene bisimide derivatives with either electron-withdrawing or electron-donating groups have been reported in the literature, including: (1) cyano-substituted PBIs [56,57]; (2) nitro-substituted PBIs [58,59,60]; (3) perfluoroalkyl-substituted PBIs [61,62]; (4) aryl-substituted PBIs [63,64]; (5) ferrocenyl-substituted PBIs [65,66]; (6) boryl-substituted PBIs [67]; (7) alkyl-substituted PBIs [68]; (8) hydroxy-substituted PBIs [69,70]; (9) alkoxy-substituted PBIs [71,72,73,74,75]; (10) amino-substituted PBIs [76,77]; (11) alkylamino-substituted PBIs [78,79,80]; (12) pyrrolidinyl-substituted PBIs [81,82,83]; (13) piperidinyl-substituted PBIs [84,85,86]; *etc*.

To date, a useful strategy for introducing substituents onto the PBIs core is bromination of perylene dianhydride. Subsequently, nucleophilic substitutions and metal-catalyzed cross-coupling reactions can then be executed. However, these reactions are usually accompanied by extensive debromination [78] and stringent reaction conditions, such as high temperatures and absence of water and oxygen. In an effort to expand the scope of PBI-based chromophores available for designing systems for colorful dyes and charge transport, we synthesized a series of purple dyes based on 1-aminoperylene bisimides [76]. We now report on the introduction of different long alkyl chains of 1-aminoperylene bisimide (**2**) affording chromophores (**1a**–**1c**) that are intense green in color and that readily undergo two quasi-reversible one-electron oxidations and two quasi-reversible one-electron reductions.

## 2. Experimental Section

### 2.1. General

The starting materials, such as perylene-3,4,9,10-tetracarboxyldianhydride, cyclohexylamine, acetic acid, *N*-methyl-2-pyrrolidinone (NMP), cerium (IV) ammonium nitrate (CAN), tin (II) chloride dihydrate (SnCl_2_.2H_2_O), tetrahydrofuran (THF), sodium hydride (NaH), 1-iodohexane (C_6_H_13_I), 1-iodododecane (C_12_H_25_I) and 1-iodooctadecane (C_18_H_37_I) were purchased from Merck (Whitehouse Station, NJ, USA), ACROS (Pittsburgh, PA, USA) and Sigma–Aldrich (St. Louis, MO, USA). Solvents were distilled freshly according to standard procedures. Column chromatography was performed using silica gel Merck Kieselgel *si* 60 (40–63 mesh). ^1^H NMR spectra were recorded in CDCl_3_ on a Bruker 400 MHz NMR spectrometer (Palo Alto, CA, USA). Mass spectra (FAB: fast atom bombardment) were recorded on a VG70-250S mass spectrometer (Tokyo, Japan). The absorption and emission spectra were measured using a Jasco V-570 UV–Vis spectrophotometer (Tokyo, Japan) and a Hitachi F-7000 fluorescence spectrophotometer (Tokyo, Japan), respectively. Cyclic voltammetry (CV) was performed with a CH instrument (Austin, TX, USA) at a potential rate of 200 mV·s^−^^1^ in a 0.1 M solution of tetrabutylammonium hexafluorophosphate (TBAPF_6_) in dichloromethane. Platinum and Ag/AgNO_3_ electrodes were used as counter and reference electrodes, respectively.

### 2.2. Synthesis

#### 2.2.1. Perylene Bisimide (**4**)

A suspension of perylene dianhydride (900 mg, 2.3 mmol), cyclohexylamine (570 mg, 5.8 mmol) and acetic acid (500 mg, 8.3 mmol) in 50 mL of *N*-methyl-2-pyrrolidinone was stirred at 80 °C under nitrogen for 8 h. After the mixture was cooled to room temperature, the precipitate was isolated by filtration, washed with 200 mL of MeOH and dried in a vacuum. The crude product was purified by silica gel column chromatography with eluent CH_2_Cl_2_ to afford **4** (950 mg, 75%). Characterization data for **4**: ^1^H NMR (400 MHz, CDCl_3_) δ 8.64 (d, *J* = 8.0 Hz, 4H), 8.60 (d, *J* = 8.0 Hz, 4H), 5.05 (m, 2H), 2.58 (m, 4H), 1.91 (m, 4H), 1.76 (m, 6H), 1.36–1.46 (m, 6H). MS (FAB): m/z (relative intensity) 555 (M + H^+^, 100); HRMS: calculated for C_36_H_31_N_2_O_4_ 555.2284, found 555.2290.

#### 2.2.2. Synthesis of 1-Nitroperylene Bisimide (**3**)

A mixture of bisimide **4** (900 mg, 1.6 mmol), cerium (IV) ammonium nitrate (CAN) (1.2 g, 2.2 mmol), nitric acid (0.1 M, 3.0 mL) and dichloromethane (150 mL) was stirred at 25 °C under N_2_ for 2 h. The mixture was neutralized with 10% KOH and extracted with CH_2_Cl_2_. After the solvent was removed, the crude product was purified by silica gel column chromatography with eluent CH_2_Cl_2_ to afford **3** (920 mg, 95%). Characterization data for **3**: ^1^H NMR (400 MHz, CDCl_3_) δ 8.74 (d, *J* = 7.6 Hz, 1H), 8.62–8.69 (m, 4H), 8.55 (d, *J* = 8.0 Hz, 1H), 8.18 (d, *J* = 7.6 Hz, 1H), 5.00 (m, 2H), 2.54 (m, 4H), 1.91 (m, 4H), 1.76 (m, 6H), 1.34–1.47 (m, 6H); IR (KBr): 2928, 2851, 1700, 1659, 1596, 1539, 1401, 1336, 1262, 1245, 1190, 809, 743 cm^−1^; MS (FAB): m/z (relative intensity) 600 (M + H^+^, 100); HRMS: calculated for C_36_H_30_O_6_N_3_ 600.2135, found 600.2141.

#### 2.2.3. Synthesis of 1-Aminoperylene Bisimide (**2**)

Tin chloride dihydrate (5.0 g, 22 mmol) and **3** (0.9 g, 1.5 mmol) were suspended in 50 mL of THF and stirred for 20 min. The solvent was refluxed with stirring for 2 h at 80 °C. THF was removed from the rotary evaporator, and the residue was dissolved in ethyl acetate and washed with 10% NaOH solution and brine. The organic layer was dried over anhydrous MgSO_4_, and the filtrate was concentrated under reduced pressure. The crude product was purified by silica gel column chromatography with eluent ethyl acetate/n-hexane (2/3) to afford **2** (680 mg, 80%). Characterization data for **2**: ^1^H NMR (400 MHz, CDCl_3_) δ 8.62 (d, *J* = 8.0 Hz, 1H), 8.45 (d, *J* = 7.6 Hz, 1H), 8.38 (d, *J* = 8.0 Hz, 1H), 8.25 (d, *J* = 7.6 Hz, 1H), 8.18 (d, *J* = 8.0 Hz, 1H), 8.10 (d, *J* = 8.0 Hz, 1H), 7.98 (s, 1H), 5.03, (s, 2H), 4.99 (m, 2H), 2.55 (m, 4H), 1.91 (m, 4H), 1.74 (m, 6H), 1.40–1.46 (m, 6H); IR (KBr): 3346, 3240, 2926, 1694, 1653, 1372, 1338, 1260, 806, 747 cm^−1^; MS (FAB): m/z (relative intensity) 570 (M + H^+^, 100); HRMS: calculated for C_36_H_32_O_4_N_3_ 570.2393, found 570.2396.

#### 2.2.4. General Procedure for Alkylation (**1a**–**1c**)

A mixture of a solution of **2** (400 mg, 0.70 mmol), sodium hydride (97%, 100 mg, 4.00 mmol) and dry THF (50 mL) was stirred at 0 °C under N_2_ for 30 min. Alkyl iodide (1.60 mmol) was then added, and the resulting mixture was stirred for 2 h. The resulting mixture was diluted with 15 mL of water and extracted with CH_2_Cl_2_. The crude product was purified by silica gel column chromatography with eluent ethyl acetate/*n*-hexane (1/2) to afford **1a** (**1b** or **1c**) in an 85% yield. Characterization data for **1a**: ^1^H NMR (400 MHz, CDCl_3_) δ 9.32 (d, *J* = 8.0 Hz, 1H), 8.49–8.53 (m, 2H), 8.48 (s, 1H), 8.31–8.39 (m, 3H), 5.02 (m, 2H), 3.40 (m, 2H), 3.06 (m, 2H), 2.55 (m, 4H), 1.90 (m, 4H), 1.62–1.79 (m, 8H), 1.46 (m, 4H), 1.16–1.30 (m, 16H), 0.77 (t, *J* = 6.4 Hz, 6H); ^1^^3^C NMR (100 MHz, CDCl_3_) δ 164.07, 164.06, 163.99, 163.86, 150.56, 135.47, 134.63, 133.53, 131.41, 130.61, 129.11, 128.95, 128.10, 126.98, 126.94, 126.93, 124.80, 124.03, 123.44, 123.19, 122.76, 122.11, 121.30, 121.19, 54.04, 53.82, 52.55, 31.44, 29.67, 29.18, 29.11, 27.47, 26.89, 26.59, 25.51, 22.52, 13.89; MS (FAB): m/z (relative intensity) 738 (M + H^+^, 100); HRMS: calculated for C_48_H_56_O_4_N_3_ 738.4271, found 738.4277. Selected data for **1b**: ^1^H NMR (400 MHz, CDCl_3_) δ 9.29 (d, *J* = 8.0 Hz, 1H), 8.47–8.52 (m, 2H), 8.46 (s, 1H), 8.29–8.43 (m, 3H), 5.01 (m, 2H), 3.38 (m, 2H), 3.05 (m, 2H), 2.56 (m, 4H), 1.90 (m, 4H), 1.60–1.77 (m, 6H), 1.46 (m, 6H), 1.11–1.20 (m, 40H), 0.82 (t, *J* = 6.7 Hz, 6H); ^1^^3^C NMR (100 MHz, CDCl_3_) δ 164.05, 164.03, 163.97, 163.82, 150.56, 135.44, 134.60, 133.49, 131.40, 130.57, 129.09, 128.93, 128.05, 126.95, 126.91, 126.89, 124.77, 124.01, 123.42, 123.18, 122.75, 122.00, 121.27, 121.14, 54.04, 53.81, 52.49, 31.86, 29.68, 29.56, 29.50, 29.28, 29.19, 29.11, 27.50, 27.21, 26.60, 25.51, 22.64, 14.07; MS (FAB): m/z (relative intensity) 906 (M + H^+^, 100); HRMS: calculated for C_60_H_80_O_4_N_3_ 906.6149, found 906.6141. Selected data for **1c**: ^1^H NMR (400 MHz, CDCl_3_) δ 9.37 (d, *J* = 8.0 Hz, 1H), 8.53–8.56 (m, 2H), 8.48 (s, 1H), 8.38–8.43 (m, 3H), 5.03 (m, 2H), 3.44 (m, 2H), 3.07 (m, 2H), 2.56 (m, 4H), 1.91 (m, 4H), 1.63–1.73 (m, 6H), 1.47 (m, 6H), 1.12–1.20 (m, 64H), 0.85 (t, *J* = 6.7 Hz, 6H); ^1^^3^C NMR (100 MHz, CDCl_3_) δ 164.15, 164.09, 164.05, 163.88, 150.54, 135.56, 134.75, 133.63, 131.51, 130.70, 129.19, 129.04, 128.17, 127.03, 124.87, 124.06, 123.50, 123.46, 123.44, 123.22, 122.82, 122.22, 121.34, 121.23, 54.03, 53.82, 52.61, 31.91, 29.67, 29.64, 29.58, 29.51, 29.34, 29.27, 29.19, 29.12, 27.54, 27.22, 26.59, 25.51, 22.67, 14.09; MS (FAB): m/z (relative intensity) 1074 (M + H^+^, 100); HRMS: calculated for C_72_H_104_O_4_N_3_ 1074.8027, found 1074.8019.

## 3. Results and Discussion

### 3.1. Synthesis

**Scheme 1:**
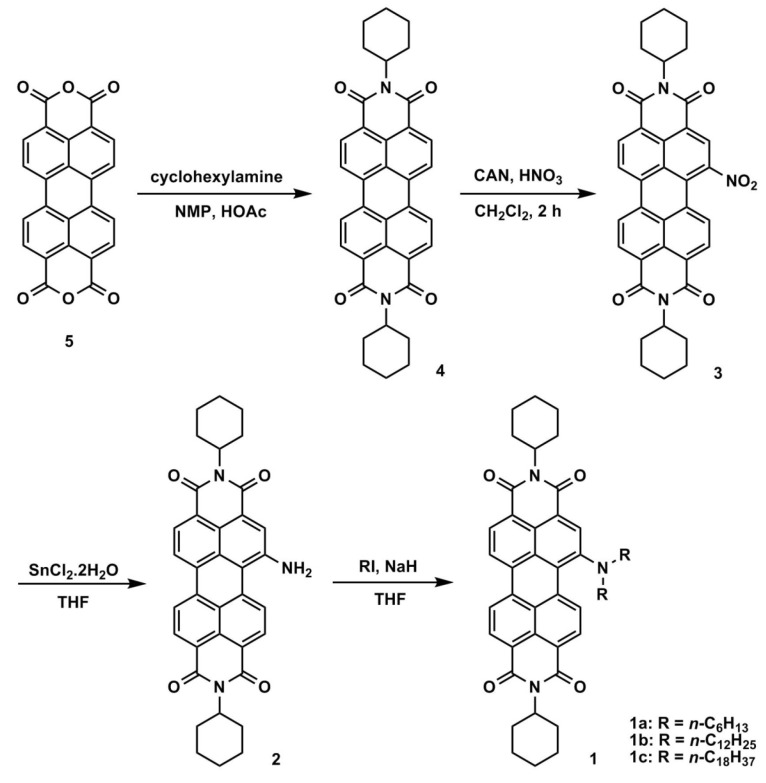
The synthetic route for **1a**–**1c**.

**Figure 1:**
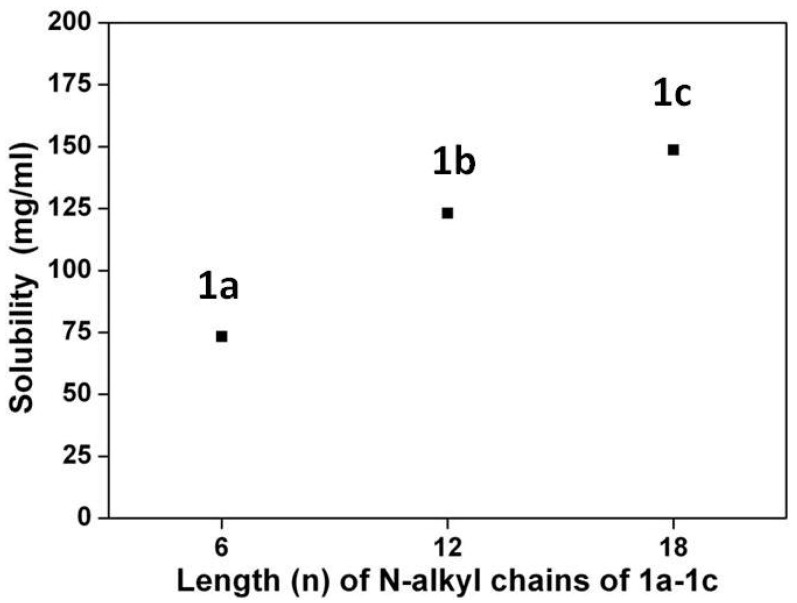
Solubility of **1a**–**1c** in dichloromethane (25 °C).

**Figure 2:**
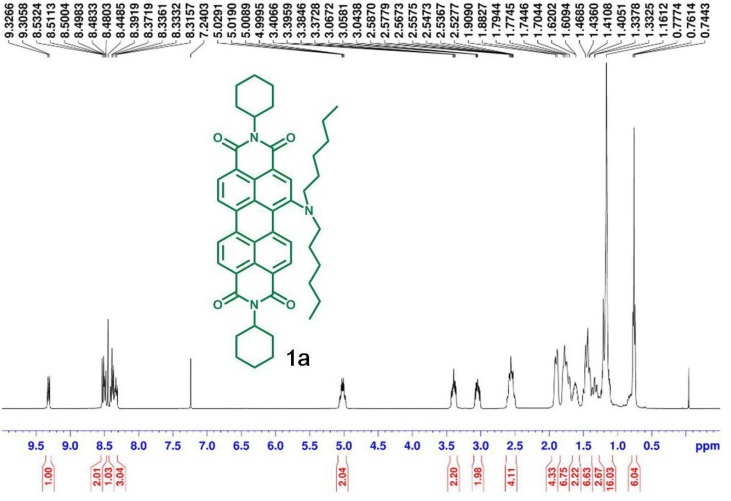
^1^H NMR (400 MHz, CDCl_3_) spectra of **1a**.

Scheme 1 depicts the chemical structures and synthetic routes of asymmetric amino-substituted PBIs (**1a**–**1c**). Synthesis starts from an imidization of perylene dianhydride (**5**) by reaction with cyclohexylamine. The mono-nitration can then be achieved by a reaction of perylene bisimide (**4**) with cerium (IV) ammonium nitrate (CAN) and HNO_3_ under ambient temperature for 2 h [58], giving **3** in high yields of *ca*. 90%. The reduction of 1-nitroperylene bisimide (**3**) by tin (II) chloride dihydrate (SnCl_2_·2H_2_O) in refluxing THF obtained 1-aminoperylene bisimide (**2**). Finally, three highly soluble perylene bisimide derivatives (**1a**–**1c**) with different *n*-alkyl chain lengths (*n* = 6, 12, or 18, Figure 1) can be prepared by the alkylation of **2** with the corresponding alkyl halides. The asymmetric structure of 1-(*N*,*N*-dialkylamino)perylene bisimides (**1a**–**1c**) can be verified by the presence of seven signals (one singlet and six doublet signals) at δ 8.3–9.4 ppm in the ^1^H NMR spectrum, which indicates that there are seven different kinds of protons in the perylene core (Figure 2). Detailed synthetic procedures and product characterization are provided in the Experimental Section.

### 3.2. Optical Properties

**Figure 3:**
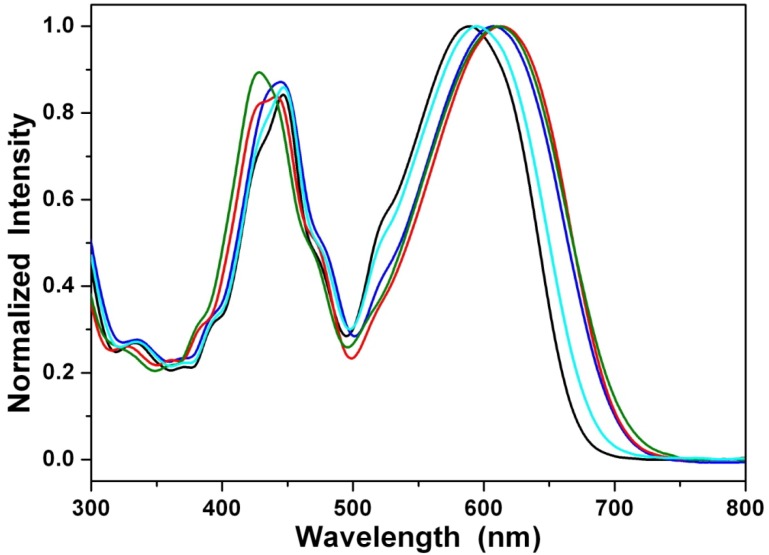
Normalized absorption spectra of **1a** in cyclohexane (black line), diethyl ether (cyan line), ethyl acetate (blue line), dichloromethane (green line) and acetonitrile (red line).

Figure 3 shows the steady-state absorption spectra of the green dye **1a** in solvents of varying polarity, and pertinent photophysical data for **1a**–**1c** are summarized in Table 1. The spectra of all of the amino-substituted PBIs (**1a**–**1c** and **2**) are dominated by broad absorption bands that cover a large part of the visible spectrum (350–750 nm). These broad bands are representative for perylene bisimide derivatives *N*-substituted at the bay-core positions, due to charge transfer absorption [78]. The longest wavelength absorption bands of **1a**–**1c** in various solvents are found to be almost the same, which indicates that different *N*-alkyl chain lengths do not significantly affect the band gap energies. Moreover, the longest wavelength absorption band of **1a**–**1c** exhibits a red shift when the solvent polarity increases (Table 1), which is consistent with the previous studies [76].

**Figure 4:**
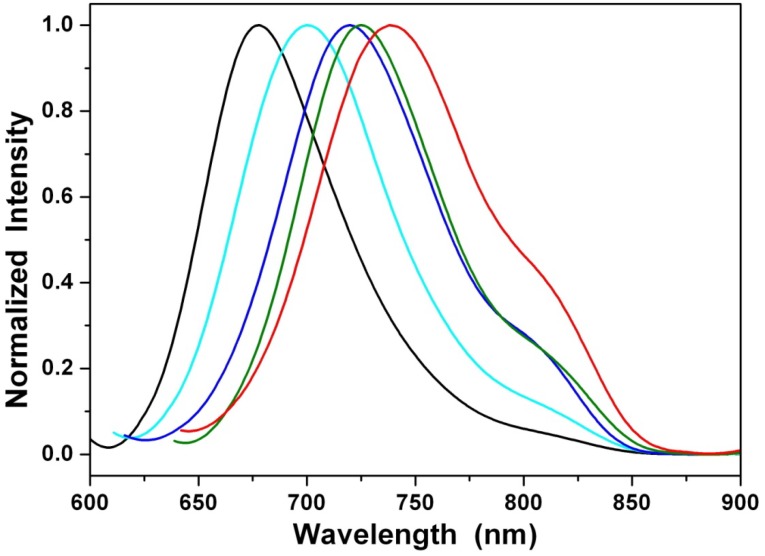
Normalized emission spectra of **1a** in cyclohexane (black line), diethyl ether (cyan line), ethyl acetate (blue line), dichloromethane (green line) and acetonitrile (red line).

The steady-state emission spectra of **1a** in different solvents of varying polarity are shown in Figure 4. Unlike the small shift in absorption spectra, the fluorescence spectra of **1a**–**1c** are largely red-shifted if there is any increase of the solvent polarity, which indicates strong intramolecular charge transfer (ICT) characteristics for the excited states of the compounds, **1a**–**1c** (Table 1). We further used the well-established fluorescence solvatochromic shift method [87] to measure the stabilization of the excited states of **1a**-**1c**. The change of magnitudes for dipole moments between ground and excited states, *i*.*e.*, 
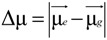
, can be estimated by the Lippert-Mataga equation and expressed as:

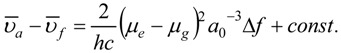
(1)
where *h* is the Planck constant, *c* is the speed of light, denotes the cavity radius in which the solute resides, *υ*_*a*_ − *υ*_*f*_ is the Stokes shift of the absorption and emission peak maximum and Δ*f* is the orientation polarizability defined as:

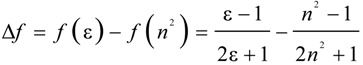
(2)


**Figure 5:**
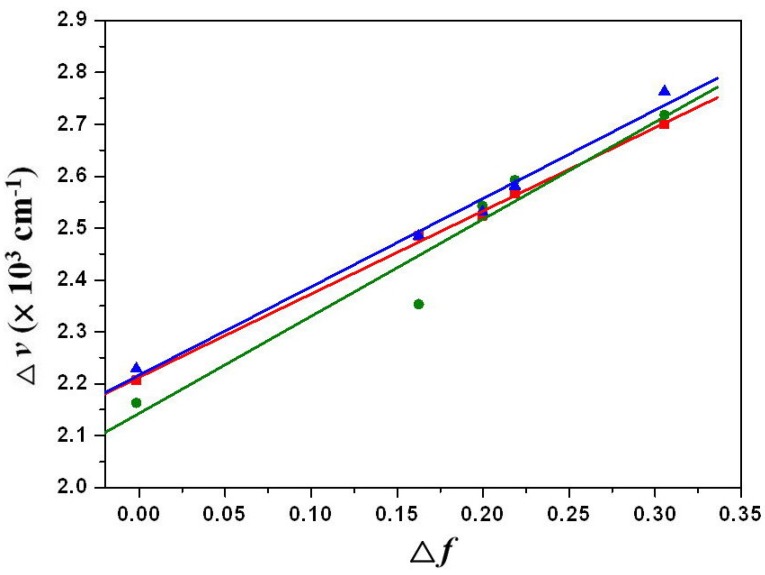
Lippert-Mataga plots for **1a** (blue line), **1b** (green line) and **1c** (red line). The solvents are (1) cyclohexane, (2) diethyl ether, (3) ethyl acetate, (4) dichloromethane and (5) acetonitrile.

The plot of the Stokes shift *υ*_*a*_ − *υ*_*f*_ as a function of Δ*f* is sufficiently linear for **1a**–**1c** (Figure 5). Accordingly, 
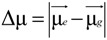
 values can be calculated as 9.0 D, 11.7 D and 12.8 D for **1a**–**1c**. These values indicate that the alkylamino-substituted PBIs (**1a**–**1c**) have larger dipole moment changes than that (7.4 D) of the amino-substituted compound (**2**).

**Table 1:** Summary of optical absorption and emission properties of **1a**–**1c** in various solvents.

1a/1b/1c	λ_abs_ (nm) ^a^	λ_em_ (nm) ^a^	Stokes shift (nm)	Φ ^b^ × 10^2^
cyclohexane	589/589/589	678/675/677	89/86/88	1.88/2.70/2.57
diethyl ether	597/601/597	700/700/701	103/99/104	0.47/0.58/0.45
ethyl acetate	608/610/610	720/722/721	112/112/111	0.24/0.31/0.29
dichloromethane	610/611/612	724/726/726	114/115/114	0.20/0.29/0.26
acetonitrile	613/614/614	738/737/736	125/123/122	0.12/0.19/0.17

^a^ Measured at 2 × 10^−5^ M; ^b^ determined with *N*,*N*’-dioctyl-3,4,9,10-perylenedicarboximide as the reference [31].

### 3.3. Quantum Chemistry Computation

**Figure 6:**
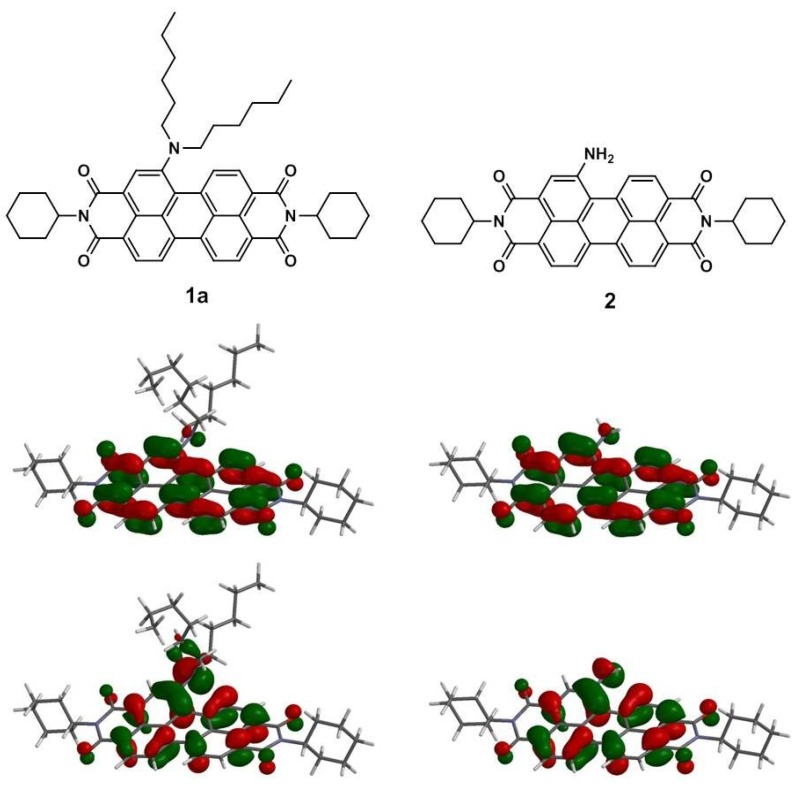
Calculated frontier orbitals for **1a** and **2**. The upper structures show the LUMOs and the lower ones show the HOMOs.

To gain more insight into the molecular structures and electronic properties of **1**–**4**, quantum chemical calculations were performed using density functional theory (DFT) at the B3LYP/6-31G** level [88,89]. The highest occupied molecular orbitals (HOMOs) and the lowest unoccupied molecular orbitals (LUMOs) of **1a** and **2** are shown in Figure 6. The HOMO of all amino-substituted PBIs (**1a**–**1c** and **2**) is delocalized mainly on the amino group and the perylene core, while the LUMO is extended from the central perylene core to the bisimide groups. The calculated and experimental parameters for perylene bisimide derivatives **1**–**4** are summarized in Table 2. It is apparent that the HOMO/LUMO energy levels of **1a**–**1c** and **2** are higher than those of **3** and **4**; this can be explained by the fact that the amino (nitro) substituent is a strong electron-donating (electron-withdrawing) group and hence increases (decreases) both the HOMO and LUMO energy levels. Additionally, the relative band gap energies estimated from the longest absorption maxima of **1**–**4** are in good agreement with the theoretical calculations (Table 2).

**Figure 7:**
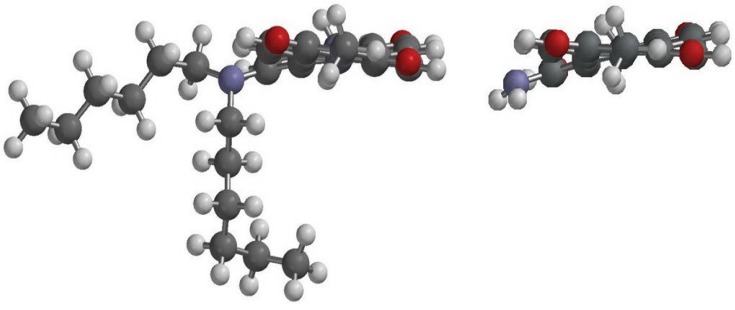
DFT (B3LYP/6-31G**) geometry-optimized structures of **1a** (**left**) and **2** (**right**) shown with the view along the long axis. For computational purposes, methyl groups replace the cyclohexyl groups at the imide positions.

DFT calculations also show that the ground-state geometries of the perylene core have different core twist angles (Figure 7), *i*.*e*., approximate dihedral angles between the two naphthalene subunits attached to the central benzene ring; these are ~9.40° and ~13.43° for **1a**, ~9.42° and ~13.45° for **1b**, ~9.45° and ~13.49° for **1c**, ~9.23° and ~17.49° for **2** and ~7.89° and ~15.87° for **3** (Table 2); and all are larger than those of **4** (~0.00°). As a whole, the core twist angles of the mono-substituted PBIs (**1**–**3**) are larger than that of the non-substituted compound (**4**).

**Table 2:** Calculated and experimental parameters for perylene bisimide derivatives.

Compound	HOMO ^a^	LUMO ^a^	*E*_g_^a^	*E*_g_^b^	μ_g_^c^	μ_e_^d^	Twisting angle (°)
**1a**	−5.48	−3.19	2.29	2.11	3.5	12.5	9.40, 13.43
**1b**	−5.48	−3.19	2.29	2.11	3.6	15.3	9.42, 13.45
**1c**	−5.47	−3.19	2.28	2.11	3.8	16.6	9.45, 13.49
**2**	−5.62	−3.21	2.41	2.24	2.7	10.1	9.23, 17.49
**3**	−6.25	−3.84	2.41	2.39	-	-	7.89, 15.87
**4**	−5.94	−3.46	2.48	2.38	-	-	0.00, 0.00

^a^ Calculated by DFT/B3LYP (in eV); ^b^ at absorption maxima (*E*_g_ = 1240/λ_max_, in eV); ^c^ ground-state dipole moment (calculated by DFT/B3LYP, in Debye); ^d^ excited-state dipole moment (in Debye).

### 3.4. Electrochemical Properties

**Figure 8:**
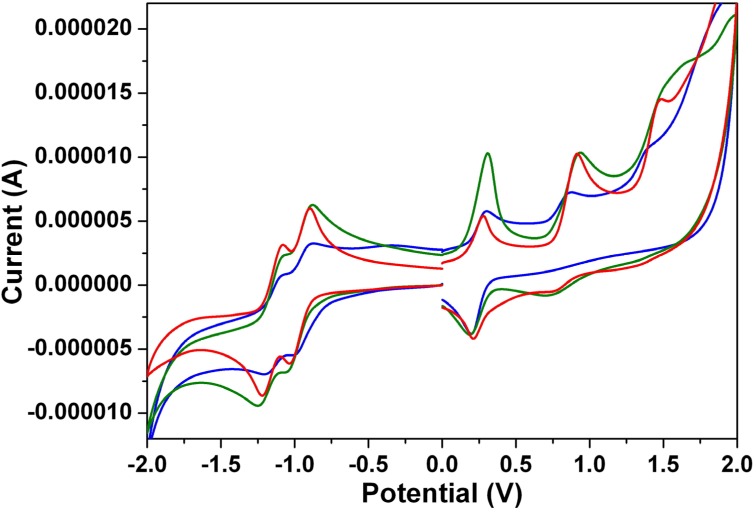
The cyclic voltammograms of **1a** (blue line), **1b** (green line) and **1c** (red line) measured in dichloromethane solution with ferrocenium/ferrocene as an internal standard, at 200 mV·s^−^^1^.

The cyclic voltammograms of **1a**–**1c** are illustrated in Figure 8. These dyes undergo two quasi-reversible one-electron oxidations and two quasi-reversible one-electron reductions in dichloromethane, which clearly indicates that all of these processes can be attributed to the successive addition or removal of electrons to the orbitals. Table 3 summarizes the redox potentials and the HOMO and LUMO energy levels estimated from cyclic voltammetry (CV) for **1**–**4**. It appears that both the first oxidation and the first reduction potentials can be shifted toward more negative (positive) values by introducing strongly electron-donating (electron-withdrawing) groups onto the perylene core, while both the HOMO and LUMO energy levels increase (decrease) with the trend.

**Table 3:** Summary of half-wave redox potentials, HOMO and LUMO energy levels for perylene bisimide derivatives.

Compound	*E*^+^_1/2_^a^	*E*^2+^_1/2_^a^	*E*^−^_1/2_^a^	*E*^2−^_1/2_^a^	HOMO ^b^	LUMO ^b^
**1a**	0.85	1.32	−1.02	−1.17	−5.47	−3.36
**1b**	0.86	1.34	−1.05	−1.21	−5.48	−3.37
**1c**	0.84	1.33	−1.01	−1.20	−5.46	−3.35
**2**	0.97	1.36	−0.97	−1.09	−5.57	−3.33
**3** ^c^	-	-	−0.19	−0.51	−6.64	−4.25
**4** ^c^	-	-	−0.46	−0.76	−6.36	−3.98

^a^ Measured in a solution of 0.1 M tetrabutylammonium hexafluorophosphate (TBAPF_6_) in dichloromethane (in V); ^b^ calculated from *E*_HOMO_ = −4.88 − (*E*_oxd_ − *E*_Fc/Fc+_), *E*_LUMO_ = *E*_HOMO_ + *E*_g_; ^c^ estimated *versus* vacuum level from *E*_LUMO_ = −4.44 − *E*_(**1**)_.

### 3.5. Stacking Behaviors of Dyes in Solution and Solid State

**Figure 9:**
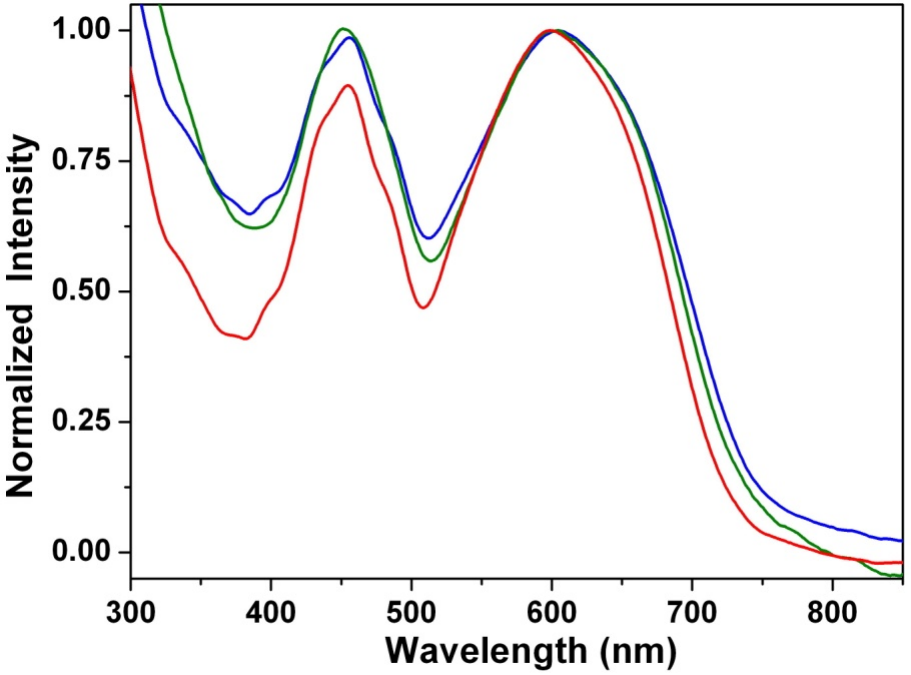
Normalized absorption spectra of **1a** (blue line), **1b** (green line) and **1c** (red line) in neat film.

Figure 9 shows the absorption spectra recorded for thin drop-cast films of **1a**–**1c**. The shapes of the absorption spectra of **1a**–**1c** in solid state and in solution are found to be virtually the same, in view of the wavelength range and peak positions. The results clearly show that it is difficult for compounds **1a**–**1c** to form π-aggregates; this can be explained by the fact that the long alkyl chains can efficiently prevent aggregation. In contrast, the absorption spectrum of the drop-cast film chromophore **2** is red-shifted, as well as broadened compared to its respective spectrum in nonpolar cyclohexane, which can be attributed mainly to intermolecular π-π interactions in the solid state [76].

### 3.6. Influence of the Acidic Condition on the Optical Properties of Dyes

**Figure 10:**
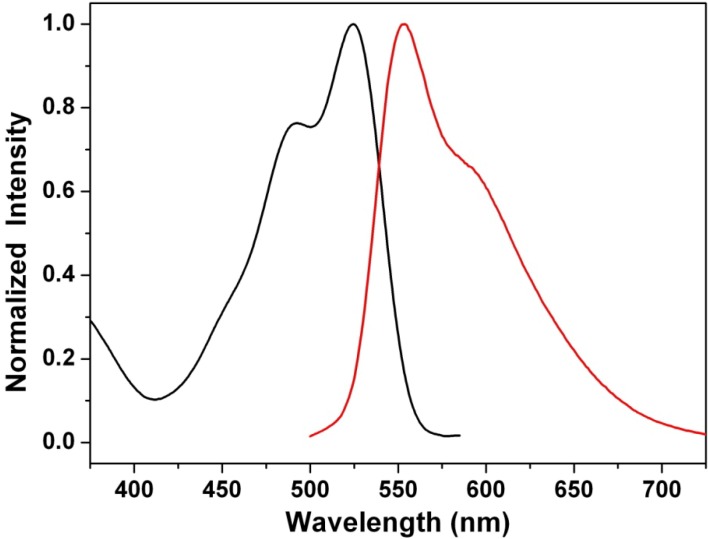
The absorption (black line) and emission (red line) spectra of **1a** in concentrated HCl.

**Scheme 2:**
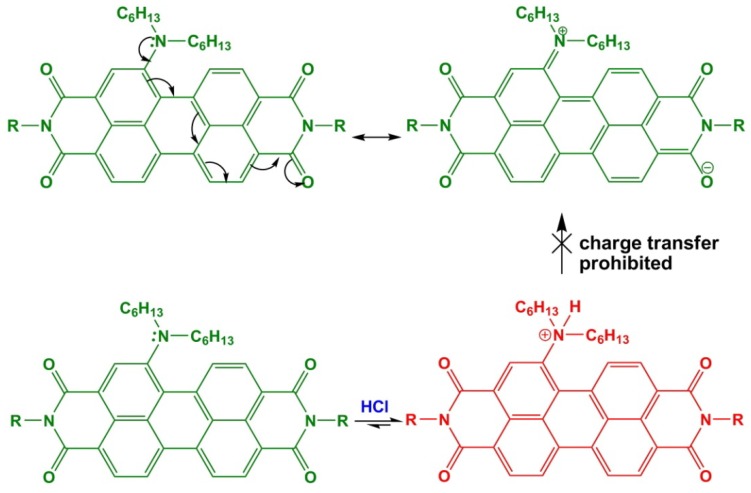
One of the possible resonance structures for **1a**, explaining the disappearance of the charge transfer absorption band in concentrated HCl.

The effects of strongly acidic conditions on the absorption and emission spectra of **1a** were also examined. Figure 10 shows the absorption and emission spectra of **1a** in concentrated HCl. The absorption spectrum of **1a** in such an acidic condition loses its typical absorption band over 590 nm and, instead, shows non-substituted PBIs centered at 520 nm, as also observed by the red color of the analyzed solution. The most likely explanation is that the nitrogen atom on the bay-core is protonated in this medium, so that charge transfer is no longer possible (Scheme 2). Moreover, the protonation-dependent change of color is completely reversible.

## 4. Conclusions

We have successfully synthesized three green dyes based on alkylamino-substituted PBIs (**1a**–**1c**). All of the new PBI dyes are soluble in most organic polar and nonpolar solvents. These molecules show a unique charge transfer emission in the near-infrared region, and the associated peaks exhibit solvatochromism. Upon excitation, they show larger dipole moment changes than that of **2**; the dipole moments of these compounds have been estimated using the Lippert-Mataga equation. Furthermore, they display reversible redox properties, as well as good optical stability. Research on their applications to organic photovoltaic cells is currently in progress.
